# Supplementary material for: Vitamins D2 and D3 Have Overlapping But Different Effects on the Human Immune System Revealed Through Analysis of the Blood Transcriptome
Source: Front Immunol. 2022 Feb 24;13:790444. doi: 10.3389/fimmu.2022.790444 (PMC8908317; doi:10.3389/fimmu.2022.790444)
Supplement: Supplementary Data File 1 — Metadata on serum concentrations of 25(OH)D2, 25(OH)D3, total 25(OH)D, PTH and calcium (albumin-adjusted) for the 97 subjects subjected to transcriptomic analysis [selected from the cohort described by Tripkovic et al. (28)]. [file DataSheet_1.zip › Supplementary Data Files/Supplementary data file 5.pdf]

Supplementary Data File 5

Networks illustrating the functional categories significantly enriched in the gene products represented by the probes significantly up-regulated or down-regulated (adj.P.Val <= 0.05) in the D_2 and D_3 treatment groups of the WE cohort (but not Placebo group).

Gene products represented by the significantly up- or down-regulated probes in the comparisons WE D2 V3 v V1, WE D3 V3 v V1 and WE P V3 v V1 from Fig. 2(a), and possessing ENTREZ identifiers, were subjected separately to functional enrichment analysis using compareCluster and processed In Cytoscape (see legend to **Fig. 4**). The complete networks for each differentially expressed group of genes are shown on pages 3 – 12.

Example of naming convention used below: **cc.WE.PD2D3.down.GOBP** are the down-regulated genes for each of the P, D2 and D3 categories, analysed by gene ontology enrichment using biological process categories.

Note: *the images are vector graphics and you can therefore zoom in to read the different functional groups*

Page	
3	cc.WE.PD2D3.down.GOBP.pdf
4	cc.WE.PD2D3.down.GOCC.pdf
5	cc.WE.PD2D3.down.GOMF.pdf
6	cc.WE.PD2D3.down.KEGG.pdf
7	cc.WE.PD2D3.down.PWAY.pdf
8	cc.WE.PD2D3.up.GOBP.pdf
9	cc.WE.PD2D3.up.GOCC.pdf
10	cc.WE.PD2D3.up.GOMF.pdf
11	cc.WE.PD2D3.up.KEGG.pdf
12	cc.WE.PD2D3.up.PWAY.pdf

Supplementary Data File 5 (continuation of legend)

Networks illustrating all the functional categories significantly enriched (p.adjust < 0.01) in analysis of the gene products represented by the probes significantly up- or down-regulated (adj.P.Val < 0.05) in the White European (WE) cohort treatment groups. The thickness of lines (edges) drawn to connect the nodes shown increases with increasing significance (p.adjust-values). This aimed to identify functional categories that are more extensively affected by D_2, or by D_3, or by both D_2 and D_3, than by the placebo, and took into consideration the changes occurring in the placebo treatment group during the period of the study. Network names correspond to the worksheets in the excel spreadsheet **Supplementary Data File 4** summarising the comparative functional enrichment analysis results, from which these networks were constructed.

Thus:

- Names ending GOBP contain results from Gene Ontology analysis using the Biological Process subontology.
- Names ending GOCC contain results from Gene Ontology analysis using the Cellular Compartment sub-ontology.
- Names ending GOMF contain results from Gene Ontology analysis using the Molecular Function sub-ontology.
- Names ending KEGG contain results from KEGG pathway analysis.
- Names ending PWAY contain results from Reactome pathway analysis.

- Names commencing cc.WE.PD2D3.down contain results from the analysis of lists of down-regulated genes from the White European D2, D3 or Placebo (P) treatment groups.

- Names commencing cc.WE.PD2D3.up contain results from the analysis of lists of up-regulated genes from the White European D2, D3 or Placebo (P) treatment groups.

- Names containing “down” = down-regulated in V3 compared to V1 in the treatment groups.

- Names containing “up” = up-regulated in V3 compared to V1 in the treatment groups.

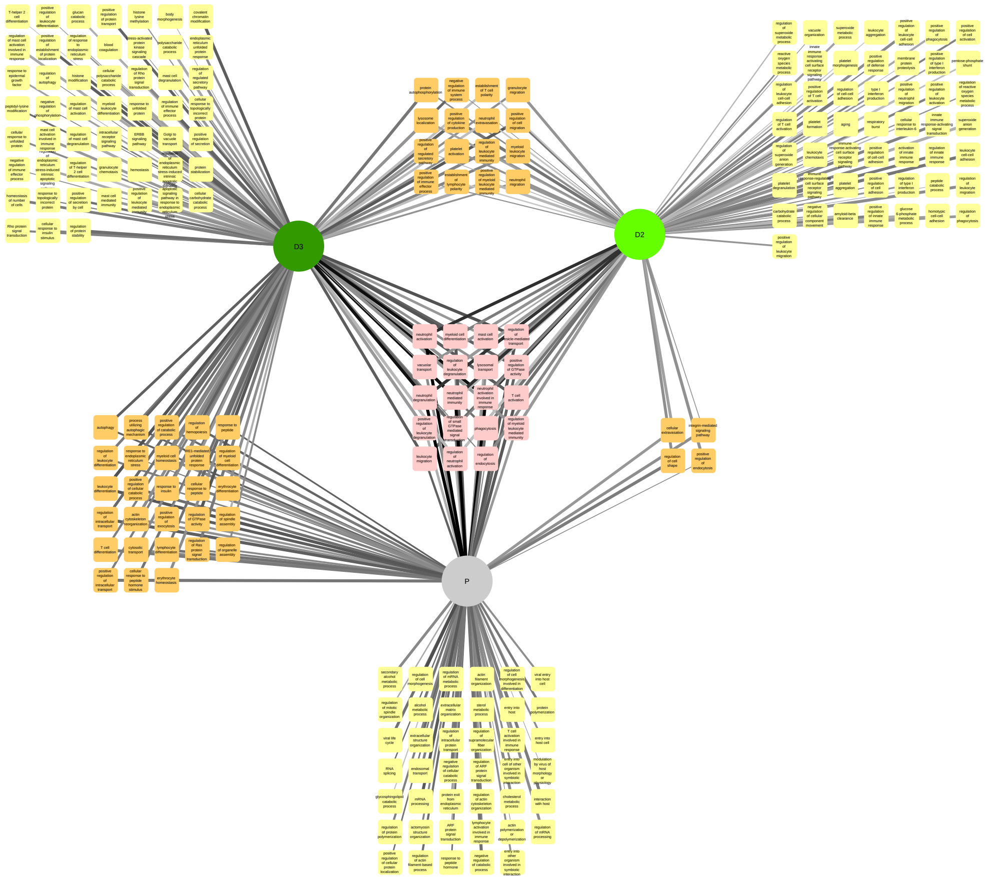

cc.WE.PD2D3.down.GOBP

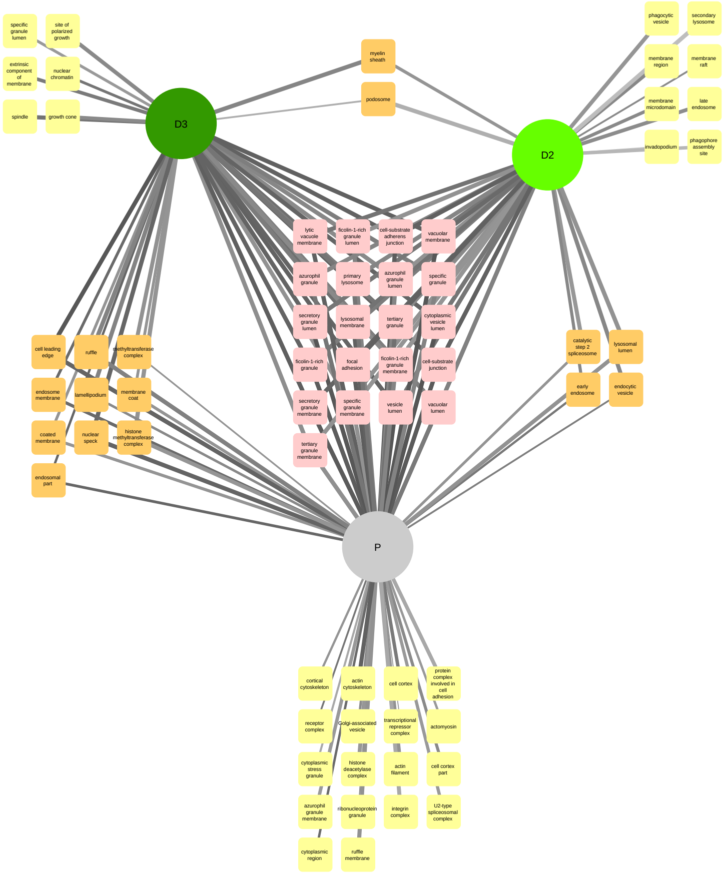

cc.WE.PD2D3.down.GOCC

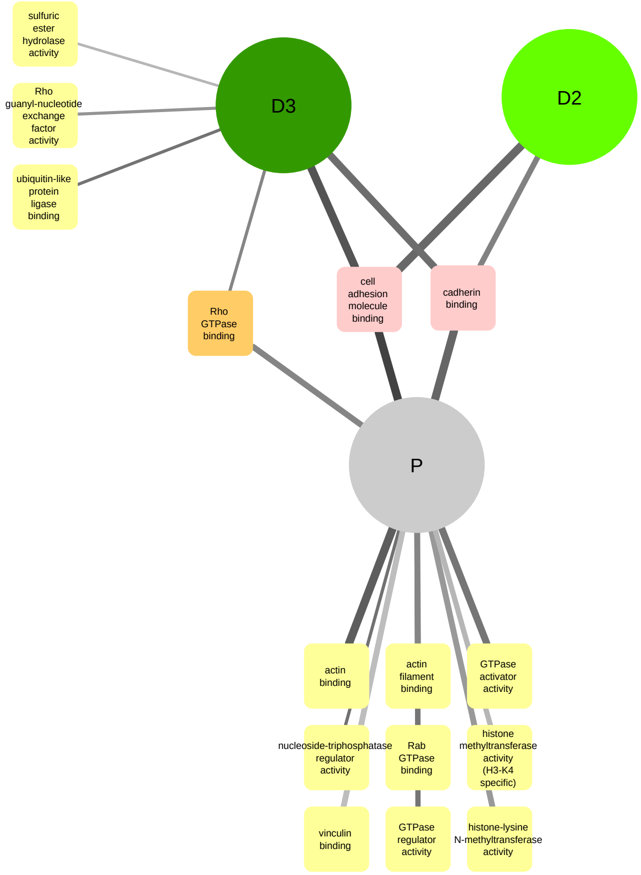

cc.WE.PD2D3.down.GOMF

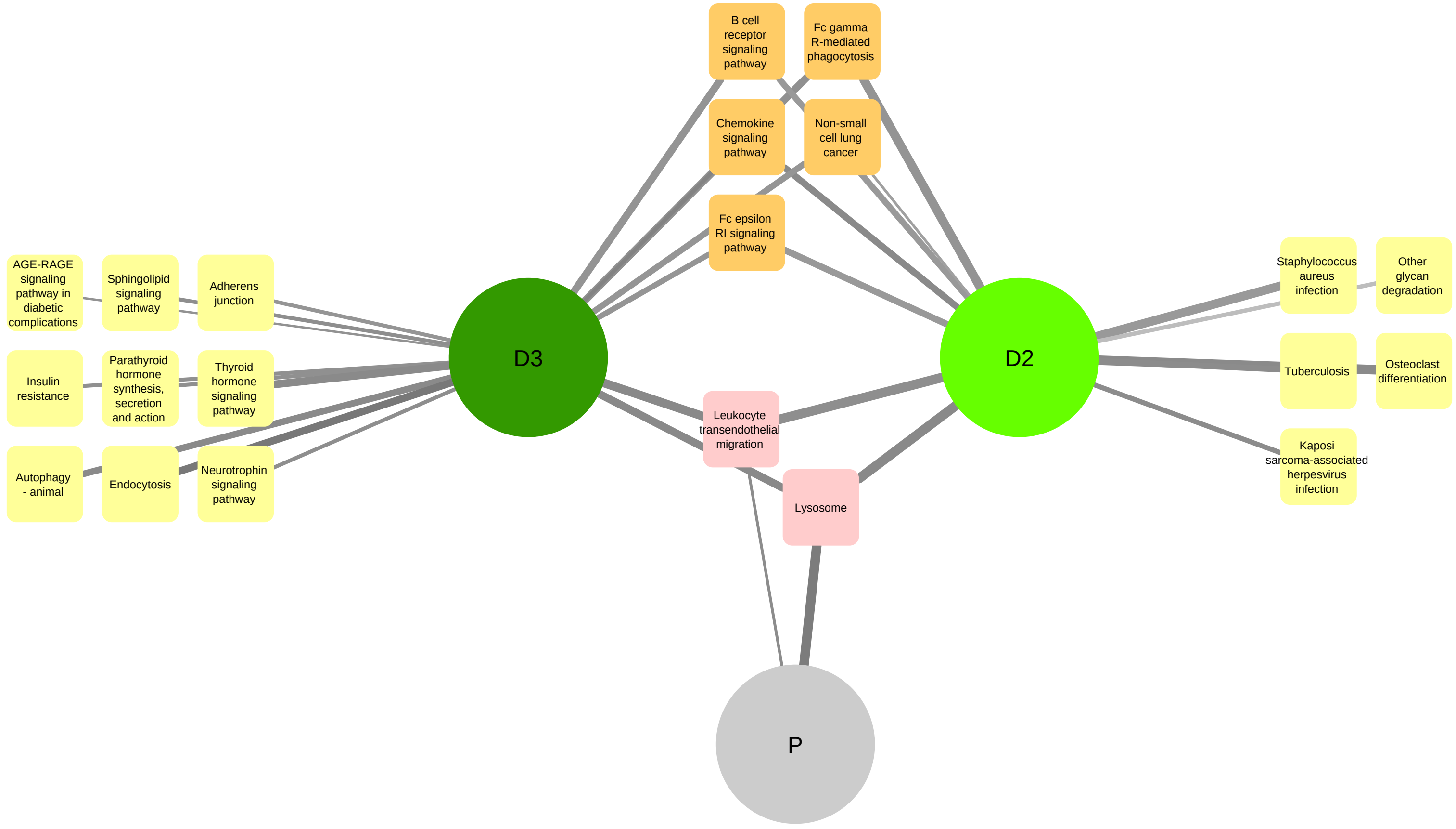

cc.WE.PD2D3.down.KEGG

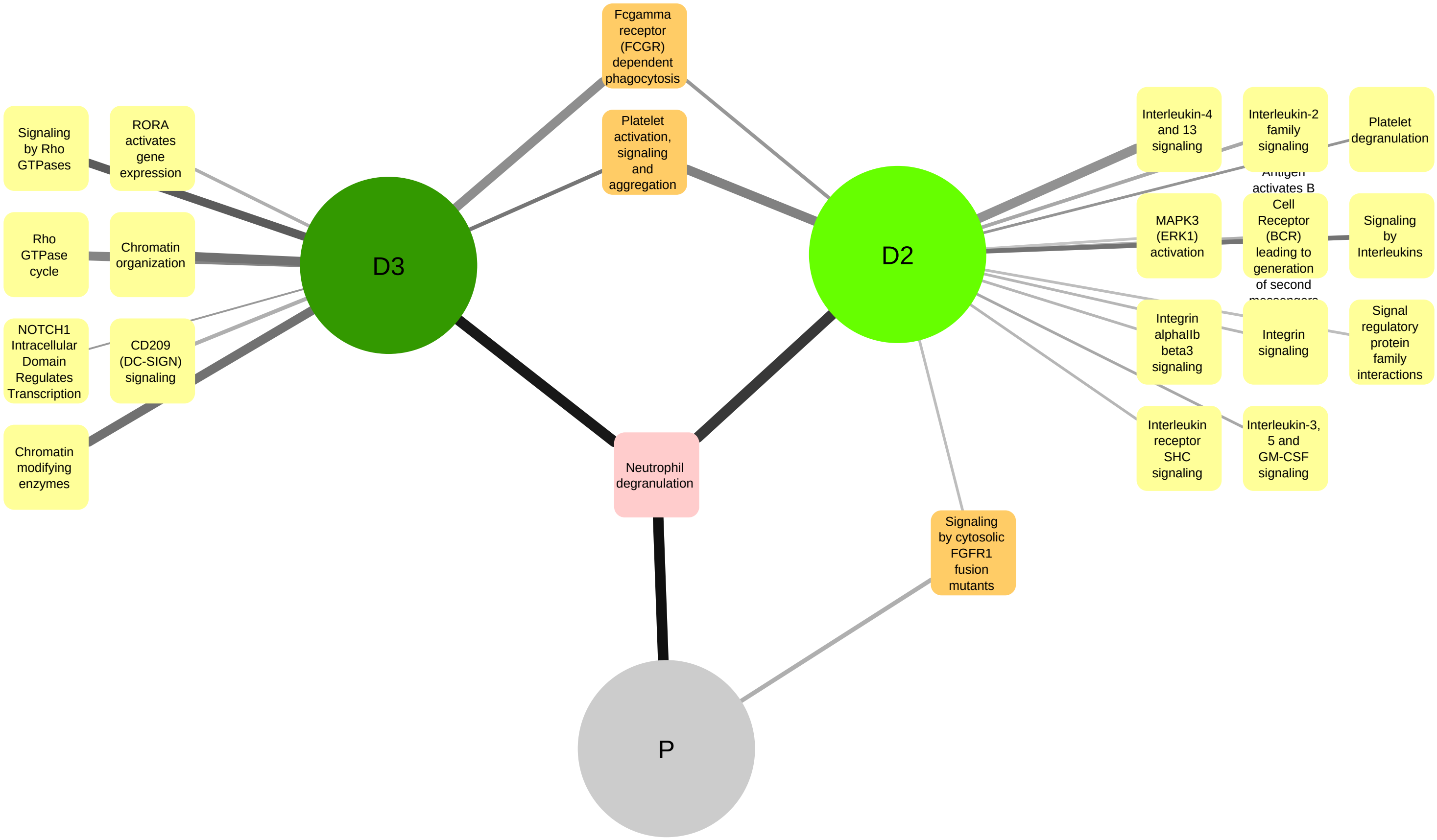

cc.WE.PD2D3.down.PWAY

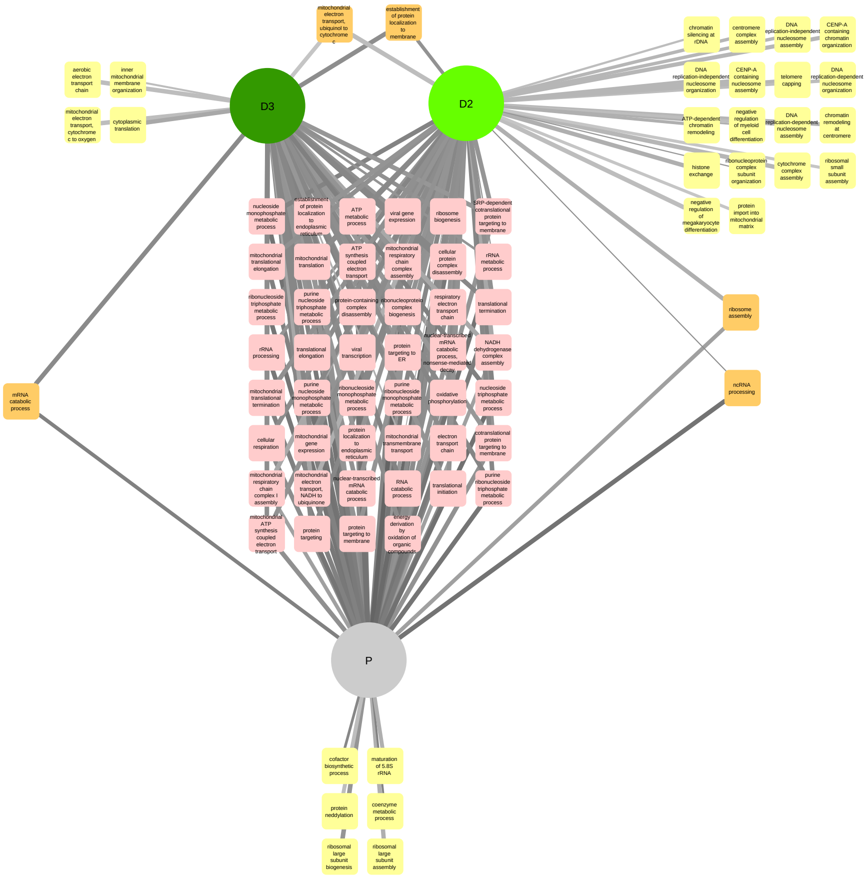

cc.WE.PD2D3.up.GOBP

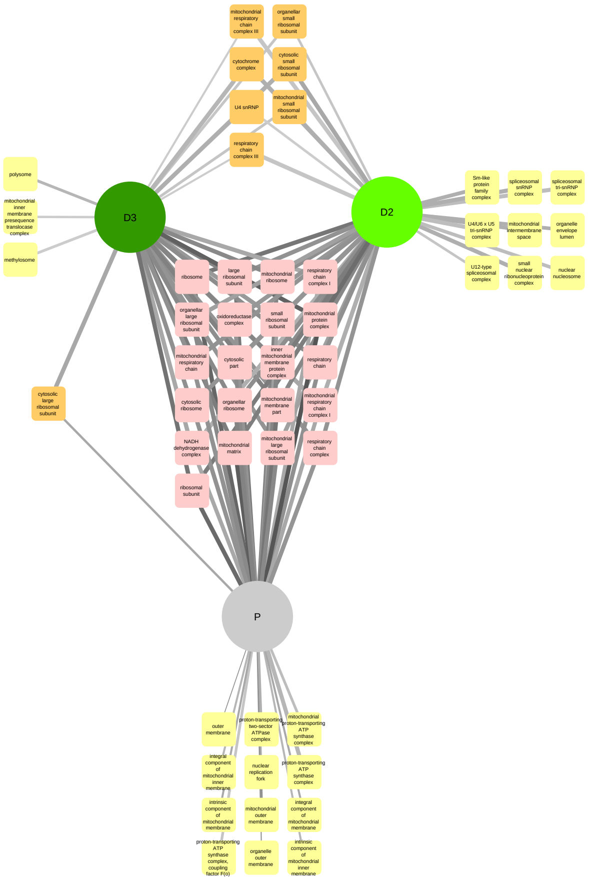

cc.WE.PD2D3.up.GOCC

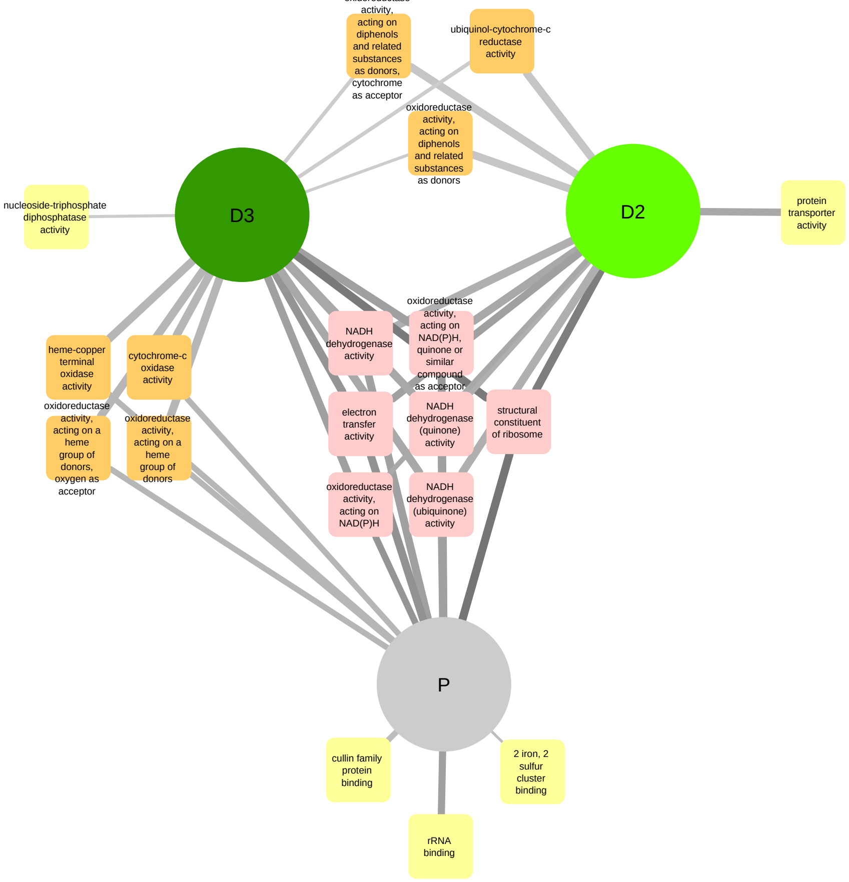

cc.WE.PD2D3.up.GOMF

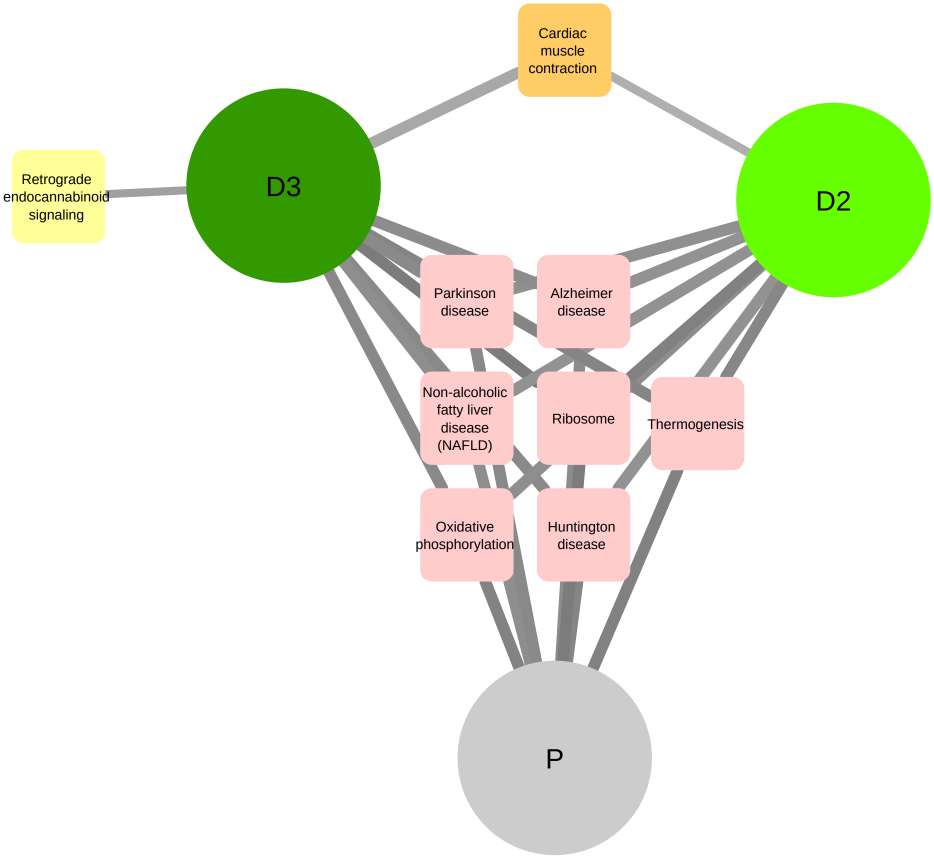

cc.WE.PD2D3.up.KEGG

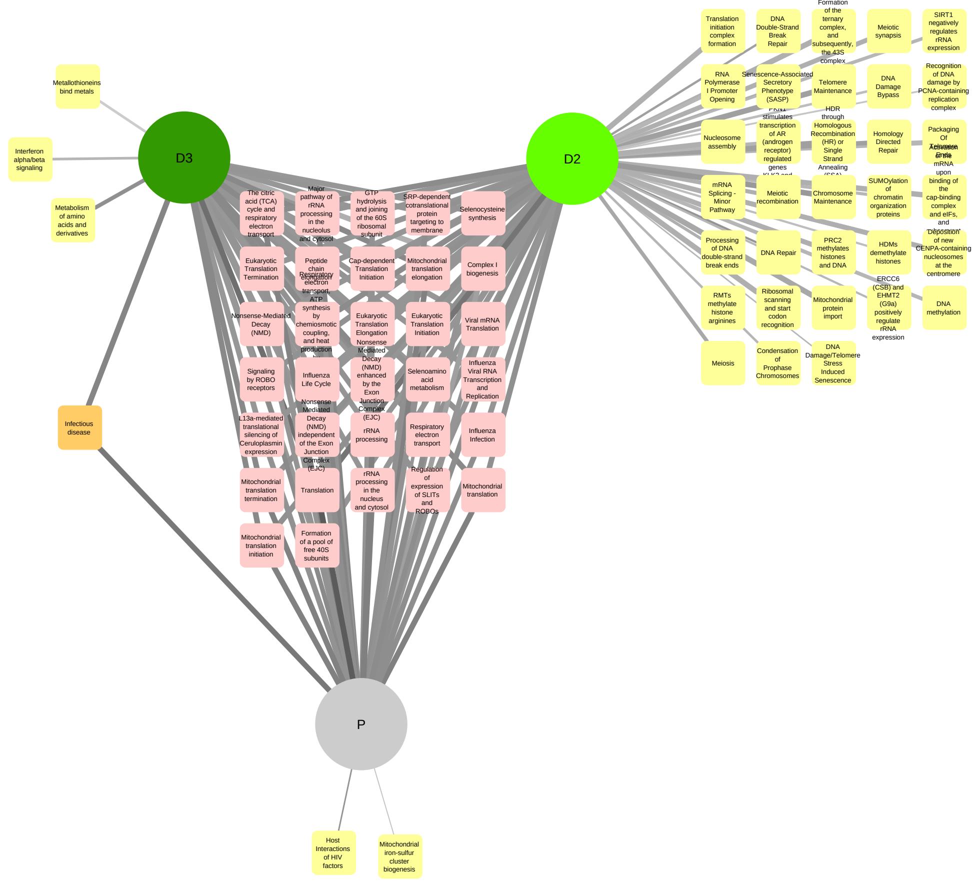

cc.WE.PD2D3.up.PWAY
