# Supplementary material for: Vitamins D2 and D3 Have Overlapping But Different Effects on the Human Immune System Revealed Through Analysis of the Blood Transcriptome
Source: Front Immunol. 2022 Feb 24;13:790444. doi: 10.3389/fimmu.2022.790444 (PMC8908317; doi:10.3389/fimmu.2022.790444)
Supplement: Supplementary file 2 [file Image_1.pdf]

a)

Probe	Systematic Name	Symbol	Gene	logFC	adj.P.Val
A_23_P79231	NM_134442	CREB1	cAMP responsive element binding protein 1	0.698193	0.021322
A_21_P0014443	THC2548652		NA	0.676465	0.001531
A_33_P3315320	NM_173478	CNTD1	cyclin N-terminal domain containing 1	0.589848	0.007448
A_23_P218706	NM_024325	ZNF343	zinc finger protein 343	-0.46626	0.025991
A_33_P3275998	NM_004703	RABEP1	rabaptin, RAB GTPase binding effector protein 1	-0.43299	0.049483

b)

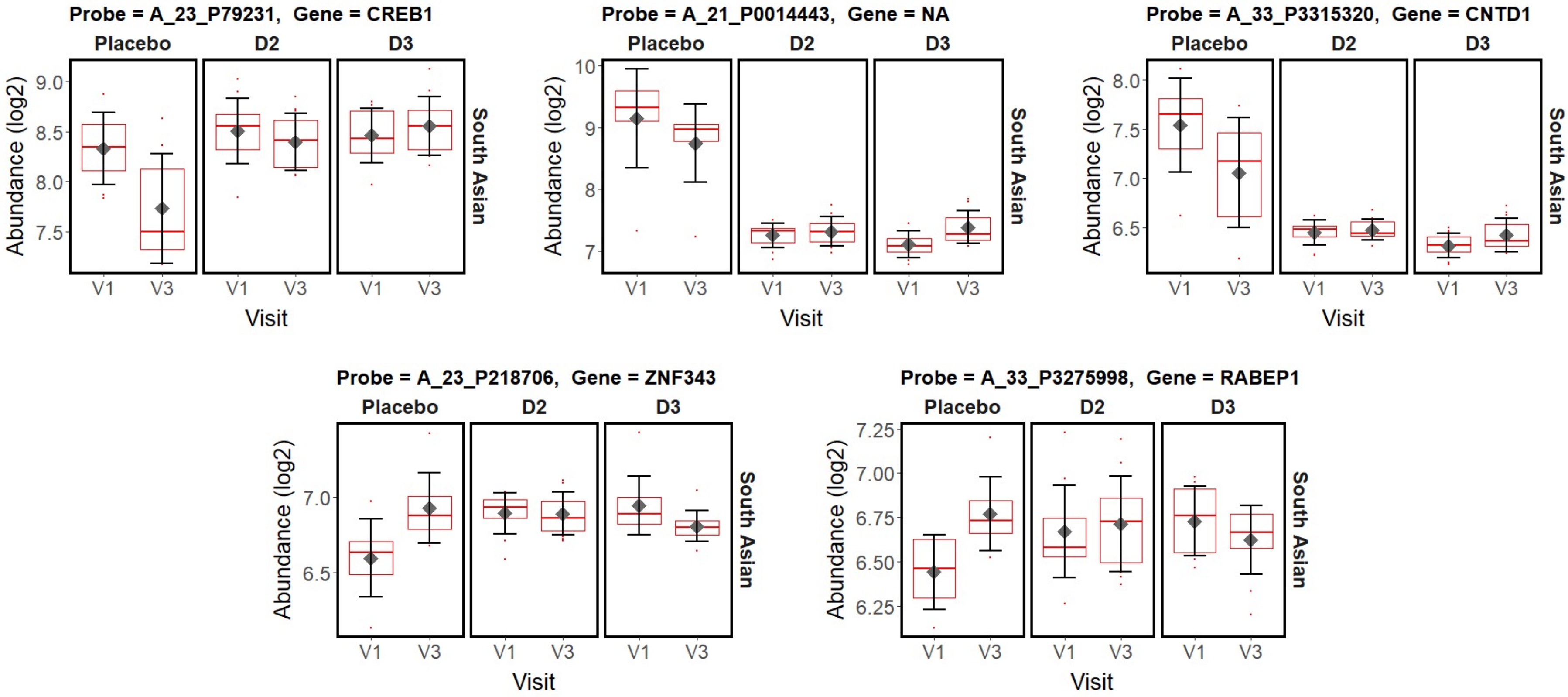

Supplementary Fig. 1. Summary of the probes identified as being significantly different in the difference in difference analysis of the SA cohort (comparison [SA D3 V3 v V1] v [SA P V3 v V1] in Fig. 2a). A) Differential expression test results. B) Probe signal abundance (log2) distribution across the experimental treatments in the SA cohort.
