# Supplementary material for: Vitamins D2 and D3 Have Overlapping But Different Effects on the Human Immune System Revealed Through Analysis of the Blood Transcriptome
Source: Front Immunol. 2022 Feb 24;13:790444. doi: 10.3389/fimmu.2022.790444 (PMC8908317; doi:10.3389/fimmu.2022.790444)
Supplement: Supplementary file 3 [file Image_2.pdf]

a)

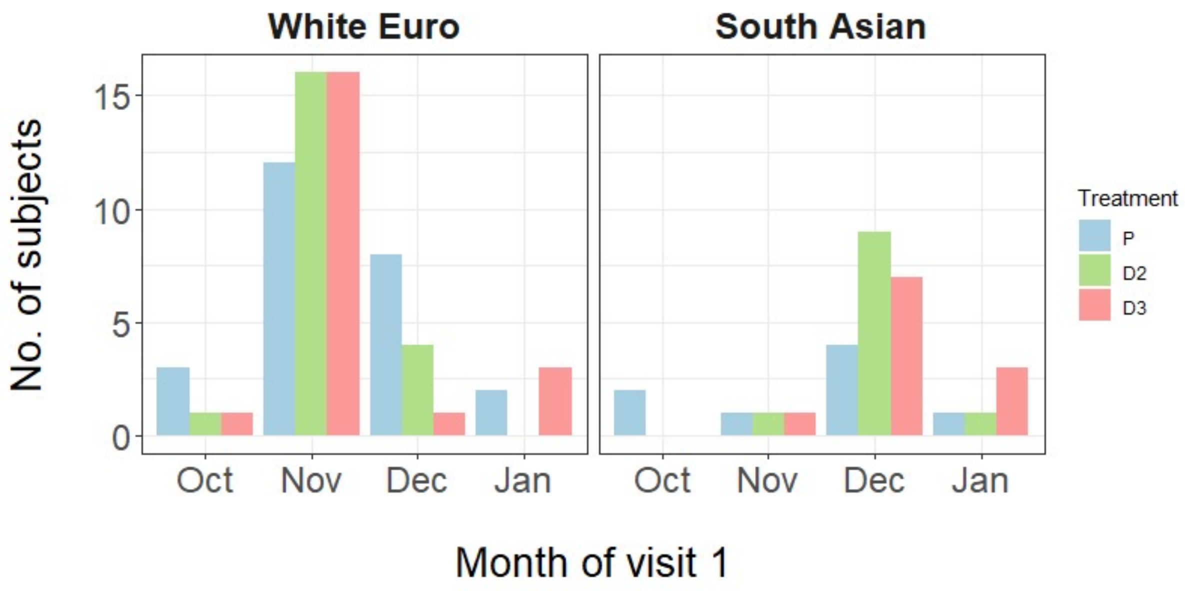

b)

	Enrichment p-value (Fisher's exact test) using the significant gene sets below		
**Comparison**	**Down V3 v V1**	**Up V3 v V1**	**All V3 v V1**
WE P	1.97E-29	2.49E-07	8.69E-35
WE D2	4.95E-28	1.14E-01	4.69E-22
WE D3	8.80E-35	7.77E-01	5.49E-27

Supplementary Fig. 2. Seasonal gene expression as a feature of the 12-week study (P = placebo, D2 = vitamin D_2, D3 = vitamin D_3). a) Summary of the timing of the V1 samples taken in the study (for each individual, V3 samples were taken 12 weeks after the V1 sample). b) Significant over-representation of the seasonal genes identified by Dopico et al (2015) *Nat Commun*, **6**, 7000 (BABYDIET dataset) in the lists of genes identified as significantly changing in the white European (WE) placebo, D_2 and D_3 treatment groups. The fisher.test function in R was used to examine the significance of the association (contingency) between classification as a seasonally expressed gene and classification as a gene significantly differently expressed in each of the nine groups shown in the table. The gene 'universe' was defined as all genes in the genome annotated with an ENSEMBL gene identifier.
