# Supplementary material for: Vitamins D2 and D3 Have Overlapping But Different Effects on the Human Immune System Revealed Through Analysis of the Blood Transcriptome
Source: Front Immunol. 2022 Feb 24;13:790444. doi: 10.3389/fimmu.2022.790444 (PMC8908317; doi:10.3389/fimmu.2022.790444)
Supplement: Supplementary file 5 [file Image_4.pdf]

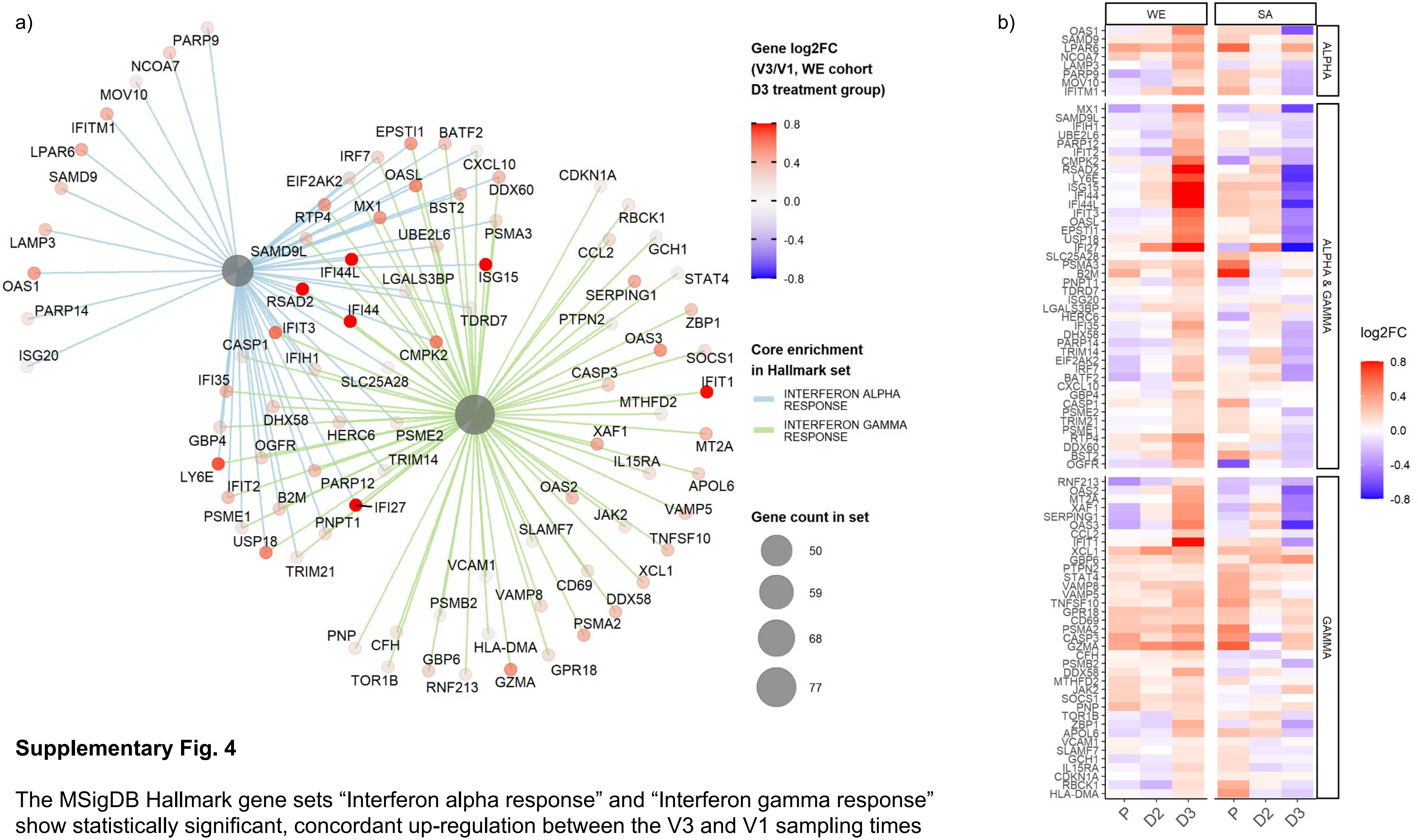

Supplementary Fig. 4

The MSigDB Hallmark gene sets “Interferon alpha response” and “Interferon gamma response” show statistically significant, concordant up-regulation between the V3 and V1 sampling times for the vitamin D_3 treatment group in the WE cohort (adjusted p-values 9.36e-15 and 1.59e-11, respectively). a) Leading edge, core genes accounting for the enrichment signals in the gene set enrichment analysis of the D_3 data. b) Heatmap showing the log2 fold change between V3 and V1 for each gene identified in a) , for all the treatment groups in both the SA and WE cohorts (P = placebo, D2 = vitamin D_2, D3 = vitamin D_3). Gene membership of the Hallmark interferon alpha and gamma response gene sets is indicated to the right. Genes shared between both sets are labelled “alpha & gamma”. Gene PARP14 and ISG20 belong to both sets, but are leading edge genes only for the alpha response.
