# Supplementary material for: Vitamins D2 and D3 Have Overlapping But Different Effects on the Human Immune System Revealed Through Analysis of the Blood Transcriptome
Source: Front Immunol. 2022 Feb 24;13:790444. doi: 10.3389/fimmu.2022.790444 (PMC8908317; doi:10.3389/fimmu.2022.790444)
Supplement: Supplementary file 7 [file Image_6.pdf]

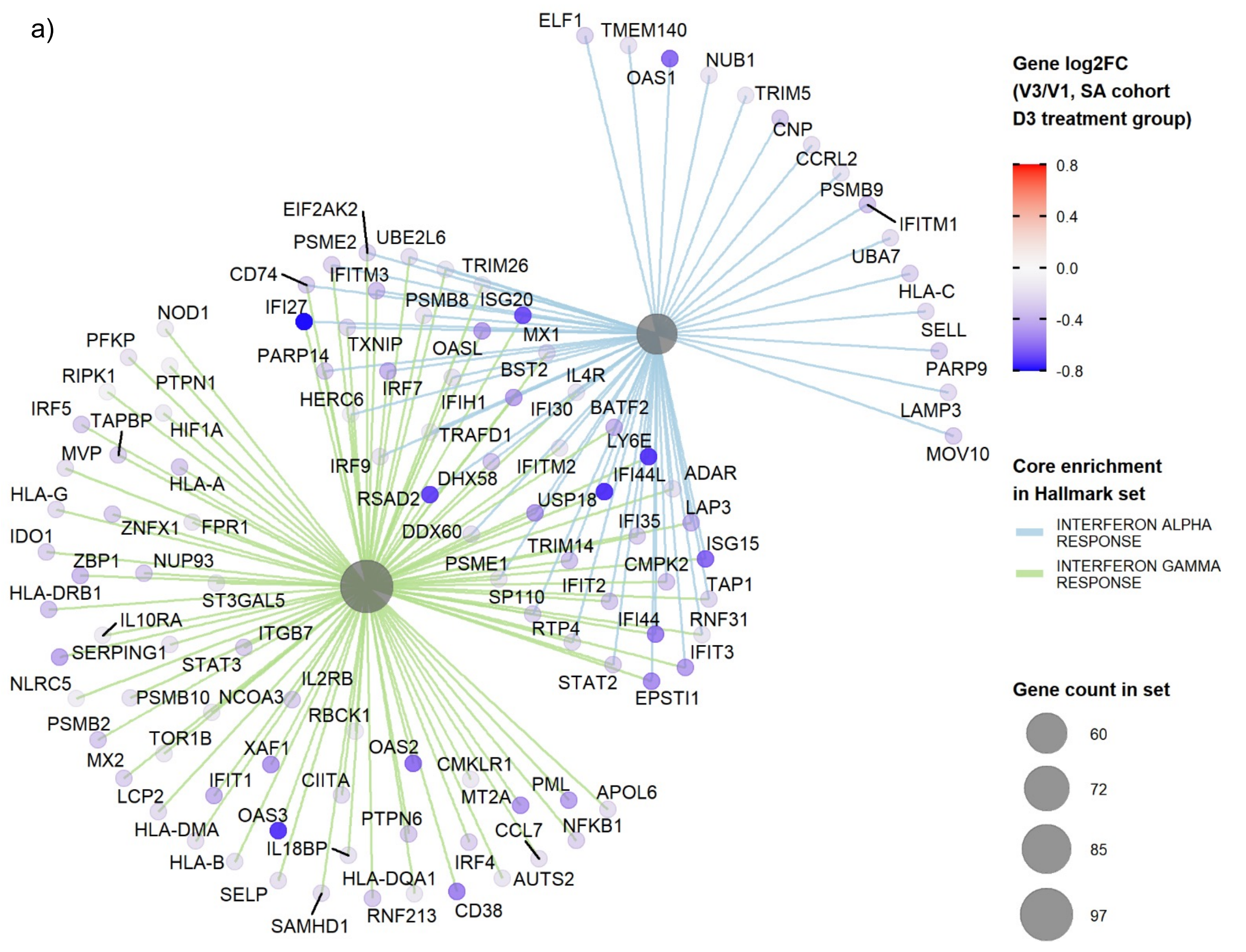

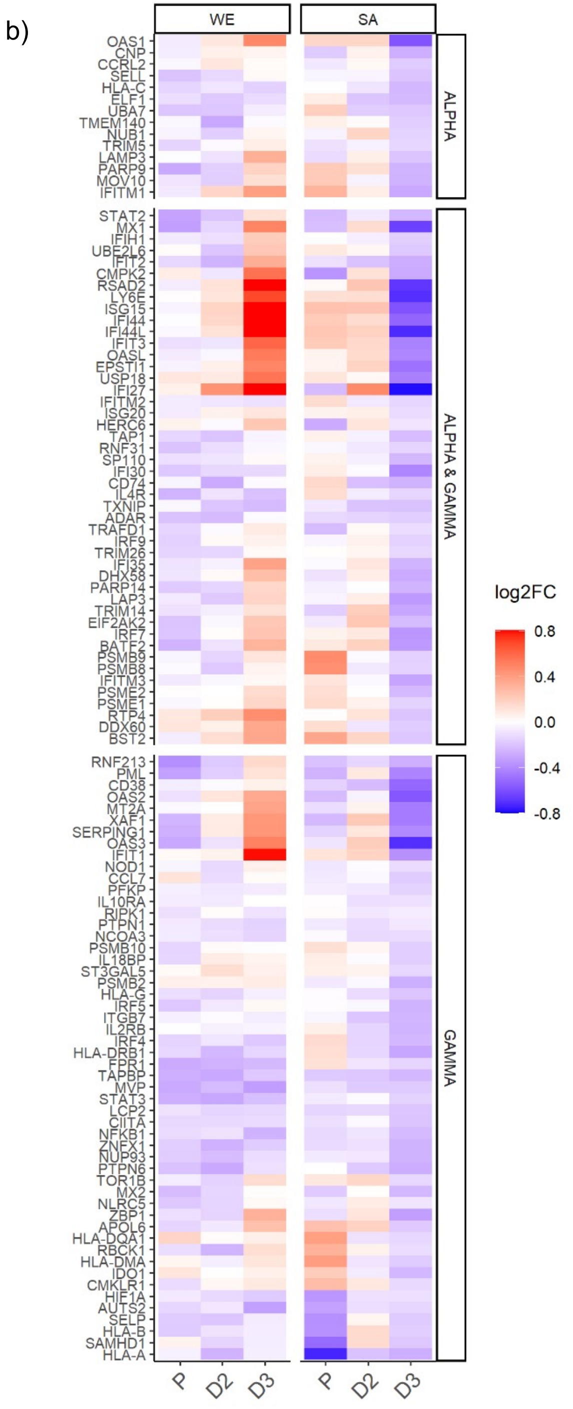

Supplementary Fig. 6

The MSigDB Hallmark gene sets “Interferon alpha response” and “Interferon gamma response” show statistically significant, concordant down-regulation between the V3 and V1 sampling times for the vitamin D_3 treatment group in the SA cohort (adjusted p-values 1.34e-09 and 8.45e-09 respectively). a) Leading edge, core genes accounting for the enrichment signals in the gene set enrichment analysis. b) Heatmap showing the log2 fold change between V3 and V1 for each gene identified in a) , for all the treatment groups in both the SA and WE cohorts (P = placebo, D2 = vitamin D_2, D3 = vitamin D_3). Gene membership of the Hallmark interferon alpha and gamma response gene sets is indicated to the right. Genes shared between both sets are labelled “alpha & gamma”. Gene PSMB8 belongs to both sets, but is a leading edge gene only for the alpha response.
