# Supplementary material for: Vitamins D2 and D3 Have Overlapping But Different Effects on the Human Immune System Revealed Through Analysis of the Blood Transcriptome
Source: Front Immunol. 2022 Feb 24;13:790444. doi: 10.3389/fimmu.2022.790444 (PMC8908317; doi:10.3389/fimmu.2022.790444)
Supplement: Supplementary file 4 [file Image_3.pdf]

PCA analysis of the 97 participants – V3 vs V1

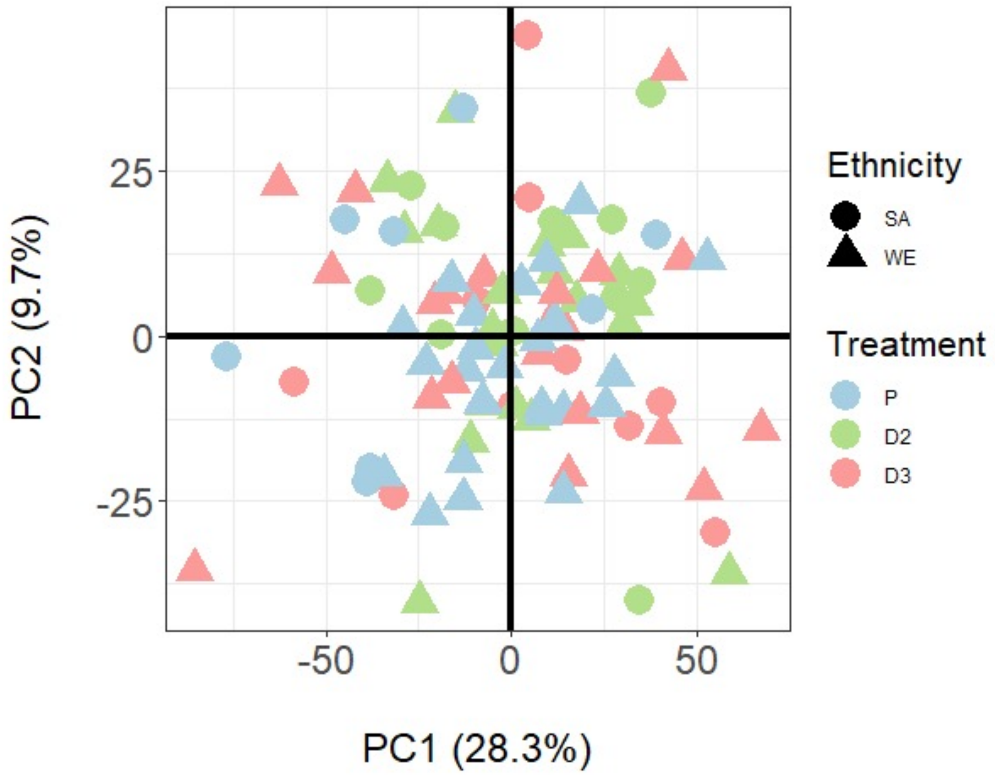

Supplementary Fig. 3.

Principal component analysis of the transcriptome data for the 97 subjects passing quality control. For each subject participant, a value defined as V3 – V1 was calculated for all the microarray gene probes in the normalised data by subtracting the abundance of each probe signal in the V1 sample from the abundance of the same probe signal in the V3 sample. The resulting matrix of values was analysed using the pca function in the R package pcaMethods (Stacklies, W., Redestig, H., Scholz, M., Walther, D. and Selbig, J. *Bioinformatics*, 2007, 23, 1164-1167). No obvious clustering by treatment group and/or ethnicity is observed.
